# Cell-free Stem Cell-Derived Extract Formulation for Regenerative Medicine Applications

**DOI:** 10.3390/ijms21249364

**Published:** 2020-12-09

**Authors:** Ashim Gupta, Craig Cady, Anne-Marie Fauser, Hugo C. Rodriguez, R. Justin Mistovich, Anish G. R. Potty, Nicola Maffulli

**Affiliations:** 1 General Therapeutics, Cleveland Heights, OH 44118, USA; ashim6786@gmail.com (A.G.); ccady@fsmail.bradley.edu (C.C.); justin@mistovich.net (R.J.M.); anishpotty@gmail.com (A.G.R.P.); 2 Future Biologics, Lawrenceville, GA 30043, USA; hcrodrig2112@gmail.com; 3 BioIntegrate, Lawrenceville, GA 30043, USA; 4 South Texas Orthopaedic Research Institute, Laredo, TX 78045, USA; 5 Veterans in Pain, Valencia, CA 91354, USA; 6 Bohlander Stem Cell Research Laboratory, Department of Biology, Bradley University, Peoria, IL 61625, USA; afauser@mail.bradley.edu; 7 School of Osteopathic Medicine, University of the Incarnate Word, San Antonio, TX 78235, USA; 8 Future Physicians of South Texas, San Antonio, TX 78235, USA; 9 Department of Orthopaedics, School of Medicine, Case Western Reserve University, Cleveland, OH 44106, USA; 10 Laredo Sports Medicine Clinic, Laredo, TX 78041, USA; 11 Department of Musculoskeletal Disorders, School of Medicine and Surgery, University of Salerno, 84084 Fisciano, Italy; 12 San Giovanni di Dio e Ruggi D’Aragona Hospital “Clinica Orthopedica” Department, Hospital of Salerno, 84124 Salerno, Italy; 13 Barts and the London School of Medicine and Dentistry, Centre for Sports and Exercise Medicine, Queen Mary University of London, London E1 4DG, UK; 14 School of Pharmacy and Bioengineering, Keele University School of Medicine, Stoke on Trent ST5 5BG, UK

**Keywords:** regenerative medicine, musculoskeletal injuries, osteoarthritis, stem cells, progenitor cells, growth factors, cytokines, extracellular vesicles, exosomes

## Abstract

Stem cells for regenerative medicine purposes offer therapeutic benefits, but disadvantages are still ill defined. The benefit of stem cells may be attributed to their secretion of growth factors (GFs), cytokines (CKs), and extracellular vesicles (EVs), including exosomes. We present a novel cell-free stem cell-derived extract (CCM), formulated from human progenitor endothelial stem cells (hPESCs), characterized for biologically active factors using ELISA, nanoparticle tracking analysis and single particle interferometric reflectance imaging sensing. The effect on fibroblast proliferation and ability to induce stem cell migration was analyzed using Alamar Blue proliferation and Transwell migration assays, respectively. GFs including IGFBP 1, 2, 3, and 6, insulin, growth hormone, PDGF-AA, TGF-α, TGF-β1, VEGF, and the anti-inflammatory cytokine, IL-1RA were detected. Membrane enclosed particles within exosome size range and expressing exosome tetraspanins CD81 and CD9 were identified. CCM significantly increased cell proliferation and induced stem cell migration. Analysis of CCM revealed presence of GFs, CKs, and EVs, including exosomes. The presence of multiple factors including exosomes within one formulation, the ability to promote cell proliferation and induce stem cell migration may reduce inflammation and pain, and augment tissue repair.

## 1. Introduction

During the past few decades, there has been a tremendous growth in the use of biologics for regenerative medicine applications [1,2]. Biologics currently available for clinical use include platelet rich plasma, bone marrow aspirate, lipoaspirate, amniotic allograft suspension, umbilical cord-derived Wharton’s Jelly, cord blood, and exosomes [3,4]. The efficacy of these biologics is attributed to the presence of stem cells, growth factors (GFs), cytokines (CKs), and extracellular vesicles (EVs), including exosomes [5,6].

The use of stem cells, including mesenchymal stem cells (MSCs), for clinical application in regenerative medicine, has gained substantial interest. MSCs can be obtained from several sources, including bone marrow, adipose tissue, trabecular bone, and deciduous teeth [7,8,9,10]. MSCs likely exert their therapeutic effect by migration to the sites of injury, engrafting, and interacting with other cells after administration [11]. Despite their therapeutic benefits, MSCs present several disadvantages, including establishing a reliable source with stable phenotype, genetic instability and chromosomal aberrations, intravenous administration-related toxicity caused by physical trapping of the cells in the lung microvasculature, rejection by the host, formation of ectopic tissue, and tumorigenicity [11,12,13].

The beneficial effects of MSCs may well not result from their ability to differentiate, but from their secretion of bioactive molecules such as GFs, CKs, and exosomes [14,15,16]. GFs are a heterogenous group of peptides/proteins and lipid soluble factors secreted by various cells including MSCs. GF receptor activation induces signal transduction pathways which initiate cell migration, proliferation, growth, and differentiation [17]. CKs are low molecular weight proteins responsible for regulation of inflammation, immune response, cellular differentiation, and tissue remodeling [18]. GFs and CKs frequently have overlapping and synergistic actions with immense potential in regenerative medicine [19]. These factors can act in an autocrine or paracrine manner: a single cytokine can promote the synthesis and release of additional CKs, leading to a cascade of molecules, influencing cell division, differentiation, and regeneration of various tissues and organs [6].

Exosomes are secreted by MSCs and act as paracrine mediators between MSCs and target cells, providing a regenerative microenvironment for damaged tissues [16,20,21]. Exosomes are small EVs with a diameter from 30–150 nm. They are formed from a sequential process of multivesicular body membrane remodeling. Exosomes are present in body fluids, including blood plasma, amniotic fluid, and umbilical cord-derived Wharton’s jelly [22]. MSCs-derived exosomes can recapitulate the MSC’s biological activity and can act as a cell-free therapeutic alternative to whole cell therapy with great regenerative potential [23,24,25]. The use of exosomes can offer advantages over whole cell therapy given their higher safety profile, lower immunogenicity, and inability to directly form tumors [26]. In addition, given their smaller size, exosomes can potentially migrate to target organs efficiently after injection, without getting trapped in the lung microvasculature [26,27].

Considering the benefits of cell-free GFs, CKs, and exosomes, and the disadvantages of whole cell therapy, innovative and effective cell-free products should be considered for clinical applications. We have formulated a novel cell-free, stem cell-derived extract (CCM) and characterized it for the presence of GFs, CKs, and EVs, including exosomes. We hypothesized that numerous GFs, CKs, and EVs, including exosomes, would be present in this formulation. This preliminary study describes the preparation and characteristics of this novel cell-free stem cell-derived extract.

## 2. Results

### 2.1. Enzyme-Linked Immunosorbent Assay (ELISA)

**Figure 1:**
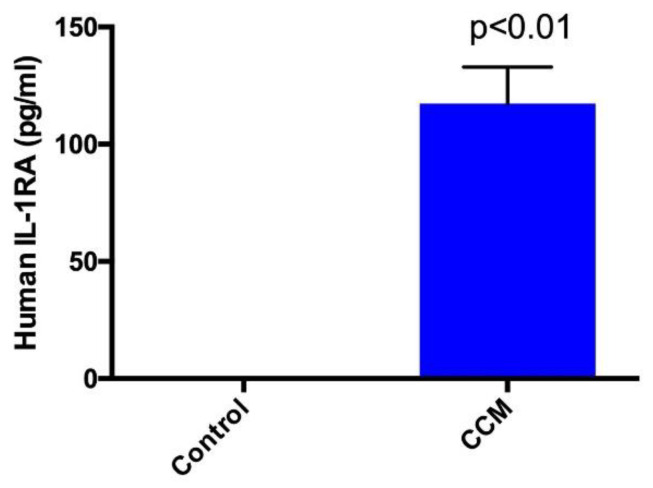
ELISA assay to determine the presence of human IL-IRA in control (media only) and cell-free stem cell-derived extract (CCM). Significantly high levels (*p* < 0.01) of IL-1RA identified in CCM compared to no detectable IL-1RA in control. Data represent mean + SEM. CCM was produced in two lots in duplicate from two vials and ELISA for IL-1RA was performed using CCM from each lot three separate times in triplicate.

GFs, including growth hormone (GH), insulin-like growth factor binding protein (IGFBP) 1, 2, 3, and 6, insulin, platelet derived growth factor-AA (PDGF-AA), transforming growth factor alpha and beta-1 (TGFα and TGFβ1), and vascular endothelial growth factor (VEGF) were detected in the formulated CCM (Table 1). In addition, IL-1RA, an anti-inflammatory cytokine was identified at significant levels in CCM relative to the control which had undetectable IL-1RA levels (Figure 1).

### 2.2. Exosome Analysis

**Figure 2:**
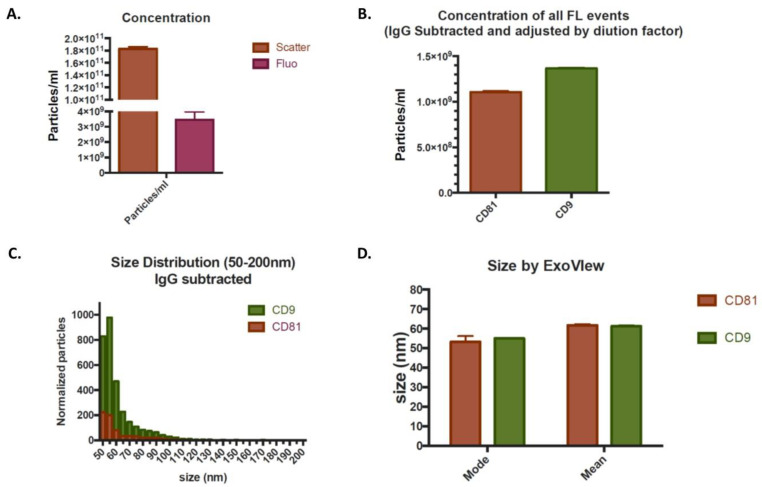
A representative nanoparticle tracking analysis showed the presence of 183 ± 3.45 billion particles/mL in the light scattering mode and 3.45 ± 0.51 billion particles in the fluorescent mode. Values are shown as mean ± standard error (**A**), and image for concentration of all fluorescent events showed presence of 1.1 billion/mL and 1.35 billion/mL CD81 and CD9 expressing particles, respectively (**B**). Representative histograms of the size distribution (**C**) and mean/mode values (**D**) of CD81- and CD9-expressing EVs measured by interferometry.

The nanoparticle tracking analysis demonstrated the presence of the average amount of 177 billion/mL of particles in the extracellular vesicle size range in the light scattering mode. Staining with the plasma membrane dye, CellMask Orange^TM^, demonstrated the presence of the average amount of 3.2 billion/mL of particles in the fluorescent mode, indicative of true membrane-enclosed particles i.e., EVs. A representative image for concentration of particles/mL determined via nanoparticle tracking analysis in the light scattering mode and fluorescent mode is shown in Figure 2A.

SP-IRIS (single particle interferometric reflectance imaging sensing) analysis with ExoView allowed high-resolution immunocapture of tetraspanin-positive (i.e., CD81/CD63/CD9) EVs and detection of the same EV markers by fluorescent counterstain, allowing for simultaneous size measurement, concentration, and phenotyping of EV subpopulations. Fluorescent marker detection by ExoView demonstrated an average amount of 1 billion/mL and 1.27 billion/mL CD81 and CD9 expressing particles, respectively. However, no CD63 positive EVs were detected. Representative image for concentration of all fluorescent events for CD81 and CD9 is shown in Figure 2B. EVs expressing CD81 and CD9 also exhibited typical size distribution with a mode of 53.34 and 55 nm as detected by interferometric measurement, which quantifies chip-bound particles in the 50–200 nm range. A representative image for size distribution and mean and mode size are shown in Figure 2C,D, respectively. Thus, expression of tetraspanins CD81 and CD9, and size of EVs <150 nm, are indicative of the presence of true exosomes.

### 2.3. Cell Proliferation

**Figure 3:**
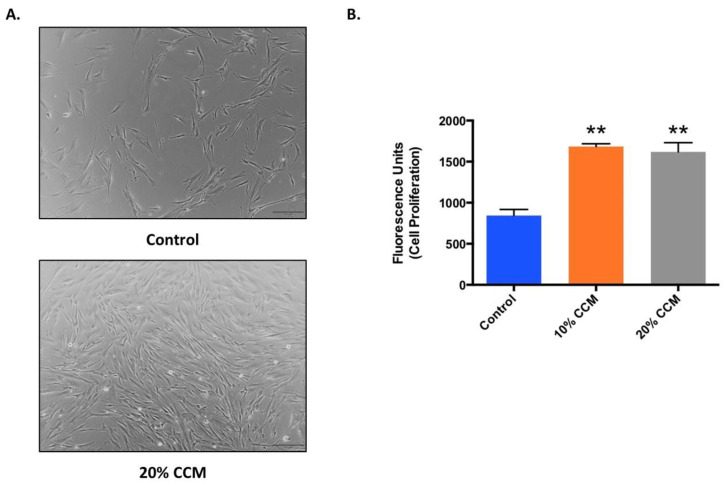
(**A**) Representative phase contrast images (size bar-100 µm) of human fibroblasts (CLL-171) treated with media only (control) and 20% CCM. (**B**) Alamar Blue assay for proliferation of CLL-171 treated with media only (control) and 10% or 20% CCM after 5 days. Data represents mean ± SEM, and ** represents significant difference (*p* < 0.0001) between CCM treated groups and control group, 5 days post-treatment with stem cell extract. CCM was produced in two lots in duplicate from two vials and cell proliferation assay was performed using CCM from each lot three separate times in triplicate.

Qualitatively, cells treated with 20% CCM showed higher density over control (media only). Representative phase contrast images of human fibroblasts treated with media only and 20% CCM is shown in Figure 3A. Similarly, the Alamar Blue proliferation assay demonstrated that cells treated with 10% or 20% CCM exhibited significantly higher (*p* < 0.0001) rate of cell proliferation compared to control after 5 days (Figure 3B).

### 2.4. Cell Migration

**Figure 4:**
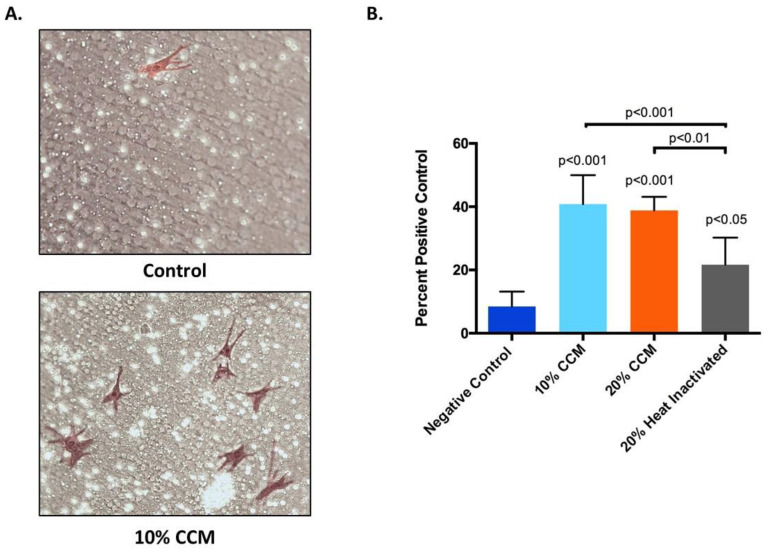
(**A**) Representative, typical images (20X) of migrated Bone marrow mesenchymal stem cells (BMSCs) on the bottom of the Transwell insert after 24 h suspension in negative control (media only) and 10% CCM. (**B**) Cell migration of BMSCs suspended in media only (negative control), 10% and 20% CCM, and heat inactivated (neutralized) 20% CCM for 24 h. Data represents mean ± SEM with significant difference between negative control versus CCM groups (*p* < 0.001) and heat inactivated CCM (*p* < 0.05); and significant reduction in inducing BMSCs migration of the heat inactivated CCM relative to the 10% CCM (*p* < 0.01) and 20% CCM (*p* < 0.001). CCM was produced in two lots in duplicate from two vials and cell migration assay was performed using CCM from each lot three separate times in triplicate.

Migration of BMSCs in response to CCM (the migration effector) was quantified as a percentage of the positive serum control using Transwell inserts. The control (media only) group induced the least number of BMSCs to migrate compared to all other groups tested. Qualitatively, images show a typical migration assay result after 24 h on the bottom of the insert with fewer migrated cells on the media only insert compared to the CCM (Figure 4A). Quantitatively, all treatment groups tested significantly increased BMSCs migration relative to the negative control, media only (Figure 4B). However, the heat inactivated CCM group had significantly lower capacity to induce BMSC migration compared to the 10% CCM and 20% CCM groups (Figure 4B).

## 3. Discussion

Over the last decade, the therapeutic use of biologics for regenerative medicine applications has led to a substantial increase in their marketing, patient demand, and clinical use [28]. While the use of biologics is promising, these treatments are still in their early stages of development [28]. Despite their widespread commercial use, there is still inadequate characterization of such biologically active formulations. In the present study, we describe the process of formulation of a novel cell-free stem cell-derived extract (CCM), and evaluated it for the presence of GFs, CKs, and EVs, including exosomes. The vital elements of regenerative medicine, namely GFs, CKs, and exosomes, are all present in this formulation. This characterization provides an initial step toward future in vivo preclinical and clinical studies to determine the safety and efficacy of CCM for regenerative medicine applications.

Numerous growth factors were identified in our CCM formulation including IGFBP 1, 2, 3, and 6, which acts as a carrier protein for IGF-1 (insulin-like growth factor-1). IGF-1 improves osteogenic differentiation of MSCs and stimulate production of extracellular matrix (ECM) [28]. Insulin was detected: this is an anabolic agent in bone, preserves and increases bone density and strength via direct and/or indirect effects on bone formation [29]. GH, which stimulates cell growth through an IGF-1 pathway and plays an essential role in cartilage regeneration, was identified [28]. PDGF-AA, which exhibits chemotactic effects towards human osteoblasts, was detected. The presence of PDGF-AA is important, as insufficient levels have been associated with cartilage degeneration [28]. TGF-α, a transforming growth factor ligand for epidermal growth factor receptor (EGFR), was also identified. EGFR promotes survival and proliferation of osteoprogenitors and plays an anabolic role in bone metabolism [28,30].

High levels of TGF-β1 were identified: TGF-β1 is a well characterized growth factor involved in the recruitment of stem/progenitor cell participation in tissue regeneration and remodeling. TGF-β stimulates cartilage repair through the stimulation of ECM production via collagen type-II and proteoglycans in chondrocytes and downregulates matrix-degrading enzymes [31]. Pre-clinical studies with TGF-β injections in the knee joint resulted in increased levels of proteoglycan of the articular cartilage. Additionally, the modulation of TGF-β signaling has been effective in several musculoskeletal pathologies including OA [32], and reported to counteract IL-1, known to induce cartilage degradation and therefore useful in cartilage repair [33].

We identified significant amounts of VEGF and its receptor VEGFR2, the main signaling receptor involved in the mediation of angiogenesis and vasculogenesis. The activation of VEGFR2 by VEGF promotes blood vessel permeability, influencing tissue repair [34]. VEGF is downregulated in patients with OA, contributing to degeneration, as VEGF is involved in new bone formation and bone tissue remodeling [28].

We also detected IL-1RA, which competitively binds IL-1 (both 1α and 1β) and blocks IL-1 mediated cellular changes. IL-1RA alleviates or prevents cytokine mediated hyperinflammatory hyperalgesia. Intra-articular injection of IL-1RA in patients with knee OA slowed its progression and improved pain and WOMAC (The Western Ontario and McMaster Universities Osteoarthritis Index) global scores [28,35]. The levels of IL-1RA in our cell-free formulation are higher compared to Wharton’s Jelly, bone marrow-derived and adipose-derived stromal cell supernatant [28,36].

The analysis of this novel CCM extract indicated the presence of true exosomes. These particles were membrane-enclosed, within the EV size range with a mode size of <150 nm, and express exosome specific tetraspanins CD81 and CD9. Interestingly, we did not observe any expression of CD63, an exosome tetraspanin. However, this may be attributed to cell specificity, as phenotype and function of stem cell-derived exosomes may vary depending on the cell source [27]. Exosomes exhibit anti-inflammatory and pro-regenerative effects to stimulate healing in different tissue types [28]. Exosomes also improve cell viability and proliferation, angiogenesis, and immunomodulation in various physiological systems [28]. Exosome uptake by cells significantly decreases the expression of pro-inflammatory genes and M1 phenotypic markers, increases cell migration and osteogenic marker expression, and exerts an osteo-immunomodulatory role in the regulation of bone dynamics [37]. Exosomes increase the secretion of cellular factors needed to hasten the healing of tendon injuries, acting via paracrine and autocrine processes [38]. In addition, exosomes stimulate repair of cartilage and proliferation of chondrocytes in osteoarthritis and help alleviate pain in knee OA [39].

The stem cell-derived extract described in the present article significantly increases the rate of fibroblasts proliferation, suggesting a potential role for improving the proliferation of cells under stress including inflammation mediated stresses. In addition, CCM significantly induces BMSCs migration in comparison to heat inactivated CCM and media only control groups. Heat inactivation denatures hydrophilic factors, significantly reducing BMSCs migration below that of the non-heated CCM. Heat inactivation did not entirely arrest BMSCs migration, indicating that heat resistant hydrophobic factors also play a role in BMSCs migration. Induction of BMSCs migration suggests a projected benefit in treatment with CCM, facilitating endogenous stem cell migration to the site of application as occurs with tissue damage, inducing chemotaxic migration of stem cells to enhance repair and recovery [40].

These results confirmed our hypotheses that GFs, CKs, and EVs, including exosomes, are present in our formulated CCM and shown to increase cell proliferation, and induce stem cell migration for tissue repair. The presence of a high density of exosomes, with multiple factors known to enhance cellular growth and differentiation with anti-inflammatory function, suggests a possible opportunity for clinical application to reduce inflammation and pain, and perhaps augment healing of orthopedic injuries. Promotion of cellular proliferation and stem cell migration can be attributed to the presence of GFs, CKs, and EVs. In accordance with previously published preclinical and clinical studies, this would indicate that the presence of a combination of these factors may prove of benefit for regenerative medicine applications [28,41]. This novel extract has equal or improved potential efficacy compared with cell-based preparations in the ability to enhance cell proliferation and induce stem cell migration. When considering possible immunogenic reactions and challenges related to cell purification, the factors identified with this novel extract, including exosomes, provide a safer alternative to cell-based treatments.

Our study is not without limitations. The ELISA kit used in our assay was limited to 40 growth factors. Further studies will be required to determine whether other growth factors and cytokines are expressed in this formulation. Further in-depth investigations will be needed to determine the functional content of the exosomes identified to determine the possible molecular implications with this extract. In addition, culturing of cells up to eight passages may also induce some age-dependent modifications. Future studies to determine the biological characteristics of the cell population including cellular aging via aging markers will be performed, as cellular aging likely affects the cellular secretome. It would also be important to assess changes in gene and cell surface protein markers to rule out possible differentiation or lineage changes that may have occurred during the development of our hPESCs cell line. Further studies will also be necessary to explore more specifically the functional mechanisms for these biological effects and the efficacy of this extract in vitro, including comparing the ability of the CCM to induce cell proliferation and migration compared to whole stem cell before exploring its potential clinical advantages via in vivo preclinical and clinical studies.

## 4. Materials and Methods

### 4.1. Cells, Reagents, and Supplies

Human progenitor endothelial stem cells (hPESCs) were obtained from Celprogen at passage 2 (catalog # 36053-05; Torrance, CA, USA; characterized by the manufacturer expressing keratin 10, 14, 15, 16, and 19, ESA, CD29, MUCI, Alcian Blue, type IV collagen, E-cadherin, CD 18/19, CD 117/cKit, and VEGFR2/KDR/FLK-1), human fibroblasts (CLL-171) from ATCC (catalog # MRC-5; Manassas, VA, USA) and bone marrow mesenchymal stem cells (BMSCs) were kindly provided by Tulane Center for Gene Therapy, Tulane University (New Orleans, LA, USA; and sourced, expanded, and characterized according to the published studies [42,43]. BMSCs were sourced from a single, normal patient and characterized for positive expression of CD 44, CD 90, CD 105 and negative expression of CD 34 with multipotency for adipogenic and osteogenic differentiation). BMSCs vials were obtained at passage 4 with 10^6^ cells/vial. These cells were further expanded for an additional 2–3 passages (P6–P7) and cryopreserved. These cells were thawed, expanded for 1–2 passages (P8–P9), and utilized for cell migration assay. Human progenitor endothelial stem cell complete media for the culture of progenitor stem cells was obtained from Celprogen (Torrance, CA, USA). Complete culture media for culturing BMSCs and fibroblast consisted of alpha MEM (Sigma, St. Louis, MO, USA), fetal bovine serum (Cytiva, Marlborough, MA, USA), Glutamax L-glutamine complex (Sigma, St. Louis, MO, USA), penicillin/streptomycin (Invitrogen, Waltham, MA, USA), and Versene (Lonza, Morristown, NJ, USA) for cell passaging. Alamar Blue Cell Proliferation Reagent was obtained from Life Technologies (Carlsbad, CA, USA), Transwell cell culture plate inserts for migration assay from Corning (Corning, NY, USA), and Transwell stain DIF Quick stain kit from MEB Inc. (San Marcos, CA, USA).

### 4.2. Cell Culture and Formulation of Novel Cell-Free Stem Cell-Derived Extract

hPESCs were cultured in serum or serum free media at 37 °C at 5% CO_2_. The cell expansion, cryopreservation, and final subculture of hPESCs for generating cells for producing CCM was completed in accordance with the manufacturer instructions and recommendations. Cells provided at passage 2 by Celprogen were initially generated from multiple human sources, shipped on dry ice at 10^6^ cells per vial. Cell expansion for banking and cryopreservation were passaged using enzyme free reagent for 3 times into multiple T-75 cell culture treated flasks with each passage initiated at 60–70% confluence. After the initial expansion process, cells were lifted in a similar manner described above, centrifuged at 750× *g*, resuspended into human endothelial progenitor cell freezing media (Celprogen #M36053-05M, Celprogen, Torrance, CA, USA), and cryopreserved under vapor phase liquid nitrogen. The resulting cells were considered a “progenitor/stem cell seed lot” and the source for all experiments and analyses performed. For the production of CCM, seed lot cells were recovered, passaged for an additional 2–3 times, and utilized to produce CCM as described below. The total number of passages including those provided by the manufacturer at passage 2 ranged from 7–8 passages. During the initial expansion and expansion prior to production of the CCM, the extract doubling time did not change from the manufacturer suggested range of 80–120 h. In addition, during cell passaging, we found no observable morphological changes to indicate cell stress, such as increases in intracellular vesicles, increases in the number of nonadherent cells or changes in general cell morphology relative to the initial cells of the seed lot. Although we did not assess the changes in gene or cell surface protein expression, we found no change in doubling time or morphology, indicating consistent estimates of confluence prior to passaging and seeding densities, increases in senescent cell density, or cell–cell contact inhibition during this process in the production of a stable hPESCs cell line. BMSCs and fibroblasts were cultured in FBS, L-glutamine in NaCl in αMEM with L-glutamine without deoxyribonucleosides media and expanded from an initial density of 50 cells/cm^2^ and cultured to confluence (>90%) in serum-based media described above. At confluence, cells were cultured under serum free conditions for 24 h at 37 °C in 5% CO_2_. After 24 h, the media was collected as conditioned media. Cells were then passaged enzyme free, pelleted at 750 g, resuspended, mechanically disrupted, and pelleted at 2000 g. The supernatant was then combined with the collected conditioned media and sterile filtered under conditions preserving growth factor and exosome functionality. All processing was performed on ice and under aseptic conditions.

### 4.3. Enzyme-Linked Immunosorbent Assay (ELISA)

Randomly selected samples from two different batches were sent to an independent laboratory, RayBiotech (Norcross, GA, USA), and were analyzed for the presence of GFs using Quantibody^®^ Human Growth Factor Array 1 kit, according to the manufacturer’s protocol. In addition, randomly selected samples from two different batches were analyzed for the presence of Interleukin 1 receptor antagonist (IL-1RA) using RayBio^®^ Human IL-1Ra enzyme linked immunosorbent assay (ELISA) kit, according to the manufacturer’s protocol.

### 4.4. Exosome Analysis

Randomly selected samples from two different batches were sent to an independent laboratory, Extracellular Vesicle Core at Children’s Hospital Los Angeles (Los Angeles, CA, USA), and were analyzed by nanoparticle tracking analysis for the presence of particles in the extracellular vesicle size range using Malvern Panalytical Nanosight NS 300. These samples were also analyzed after staining with a general fluorescent membrane marker, CellMask Orange^TM^ (Thermo Fisher Scientific, Waltham, MA, USA), as previously described [28]. The size, quantity, and surface protein characteristics of EVs were then further analyzed through single particle interferometric reflectance imaging sensing (SP-IRIS) using the ExoView platform (NanoView Biosciences, Boston, MA, USA), according to the manufacturer’s instructions and as described in the literature [44]. Briefly, ExoView uses a multiplexed microarray chip for the immuno-capture of commonly expressed EVs tetraspanin proteins-CD9, CD63, and CD81. ExoView then analyzes EVs using visible light interference for size measurements and fluorescence for protein profiling [44].

### 4.5. Cell Proliferation Assay

Alamar Blue cell proliferation assay was completed according to the manufacturer’s protocol. Briefly, human fibroblasts were plated at a density of 5000 cells/well into a 24 well plate and treated with either control (media only) or 10%, 20% CCM. Phase contrast images were taken using light microscopy (Olympus inverted fluorescent microscope with LCD DP71 Olympus digital camera, Olympus, Center Valley, PA, USA). After 5 days, cell viability assay was performed by replacing the media with the Alamar Blue reagent followed by incubation for 4 h at room temperature under light free conditions. The supernatant was then transferred to a 96 well plate and the fluorescence signal was measured at 540–570 nm excitation/580–610 nm emission using a microplate reader (Synergy LX, BioTek Instruments, Inc., Winooski, VT, USA).

### 4.6. Cell Migration Assay

Transwell migration assay was completed with passaged BMSCs across insert filters with a pore size at 8 microns against a migration effector consisting of either media only (negative control), 4% serum (positive control), 10% CCM, 20% CCM, or heat inactivated (neutralized) 20% CCM and transferred into the lower well. The insert filter membrane was then placed into the well in contact with the migration effector. Heat inactivation was accomplished by placing 20% CCM into a water bath at 100 °C for 30 min. A total of 5000 BMSCs were plated at the top of the insert and incubated at 37 °C for 24 h. After incubation, the remaining cells on the upper surface of the filter were removed with a cotton swab. Cells which migrated across the filter to the underside of the insert were fixed and stained with a Diff-Quik stain kit and counted in 5 fields at 20x for duplicate inserts. Migration was expressed as a percentage of the 4% serum positive control.

### 4.7. Statistical Analysis

CCM was produced in two lots in duplicate from two vials obtained from Celprogen. ELISA for IL-1RA was performed using CCM from each lot three separate times in triplicate and mean ± standard error of mean (SEM) values were calculated. Statistical significance was determined by unpaired *t*-test. Cell proliferation assay and migration assay were performed using CCM from each lot and experiments were repeated three separate times in triplicate and mean ± SEM values were calculated. Statistical analysis was determined using analysis of variance (ANOVA) with Tukey’s post hoc test. The results were considered significant when *p* < 0.05.

## 5. Conclusions

Our novel cell-free, stem cell-derived extract formulation showed the presence of GFs, CKs, and EVs, including a high density of exosomes, and the ability to enhance the rate of cell proliferation and induce stem cell migration. The presence of multiple factors within one formulation and their ability to promote cell proliferation and induce migration of stem cells may eventually have a clinical role in reducing inflammation and pain and augment tissue repair and regeneration without the risk of using viable cells.

## Figures and Tables

**Table 1:** Growth factors expressed in the formulated stem cell extract.

Growth Factors		Average Amount (pg/mL)
IGFBP-1	Insulin-like growth factor-binding protein-1	70
IGFBP-2	Insulin-like growth factor-binding protein-2	18.1
IGFBP-3	Insulin-like growth factor-binding protein-3	552.6
IGFBP-6	Insulin-like growth factor-binding protein-6	20.3
Insulin	Insulin	518.5
GH	Growth hormone	10.5
FGF-7	Fibroblast growth factor-7	45.5
HB-EGF	Heparin-binding EGF-like growth factor	42.2
PDGF-AA	Platelet derived growth factor-AA	58.1
TGF-α	Transforming growth factor alpha	2.4
TGFβ1	Transforming growth factor beta 1	1036.90
EG-VEGF	Endocrine-gland-derived vascular endothelial growth factor	86.6
VEGF	Vascular endothelial growth factor	30.8
VEGF R2	Vascular endothelial growth factor-receptor 2	20.6
VEGF R3	Vascular endothelial growth factor-receptor 3	300
VEGF-D	Vascular endothelial growth factor D	16.6
BDNF	Brain-derived neurotrophic factor	68.6
b-NGF	Beta-nerve Growth factor	9.5
NGF-R	Nerve growth factor receptor	21
NT-4	Neurotrophin-4	110.3

## Abbreviations

ANOVA	Analysis of variance
BMSC	Bone marrow mesenchymal stem cells
CCM	Cell-free stem cell-derived extract
CKs	Cytokines
ECM	Extracellular matrix
ELISA	Enzyme linked immunosorbent assay
EGFR	Epidermal growth factor receptor
EVs	Extracellular vesicles
GFs	Growth factors
GH	Growth hormone
IGF-1	Insulin-like growth factor-1
IGFBP	Insulin-like growth factor binding protein
IL-1RA	Interleukin 1 receptor antagonist
MSCs	Mesenchymal stem cells
OS	Osteoarthritis
PDGF-AA	Platelet derived growth factor-AA
SP-IRIS	Single particle interferometric reflectance imaging sensing
SEM	Standard error of mean
TGFα	Transforming growth factor alpha
TGFβ1	Transforming growth factor beta-1
VEGF	Vascular endothelial growth factor
